# Allergic Respiratory Diseases in the Climate Change Context: Aerobiological Tools and Projections as Preventive Actions for Pollen-Induced Allergies

**DOI:** 10.3390/medsci14040466

**Published:** 2026-08-08

**Authors:** Maria Concetta D’Ovidio, Virginia Ciardini, Andrea Lancia, Pasquale Capone, Carlo Grandi, Federico Di Rita, Lorenzo Uda, Elona Begvarfaj, Massimo D’Isidoro, Daniela Meloni, Ilaria D’Elia, Giandomenico Pace, Alcide di Sarra

**Affiliations:** 1 Department of Occupational and Environmental Medicine, Epidemiology and Hygiene, Italian Workers’ Compensation Authority (INAIL), 00078 Monte Porzio Catone, RM, Italy; a.lancia@inail.it (A.L.); p.capone@inail.it (P.C.); ca.grandi@inail.it (C.G.); 2 Italian National Agency for New Technologies, Energy and Sustainable Economic Development, 00123 Rome, Italy; massimo.disidoro@enea.it (M.D.); daniela.meloni@enea.it (D.M.); ilaria.delia@enea.it (I.D.); giandomenico.pace@enea.it (G.P.); alcide.disarra@enea.it (A.d.S.); 3 Department of Environmental Biology, Sapienza University of Rome, 00185 Rome, Italy; federico.dirita@uniroma1.it (F.D.R.); lorenzo.uda@uniroma1.it (L.U.); 4 Department of Sense Organs, Sapienza University of Rome, 00185 Rome, Italy

**Keywords:** climate change, pollen allergies, prevention, aerobiological monitoring, pollen forecasting system

## Abstract

Climate change is a global phenomenon with various impacts on human health, in many cases aggravating the effects of pollen allergies. Several studies show the effects of human-induced temperature rise and changes in meteorological patterns on pollen production and dispersion, causing earlier flowering onset, extended pollen seasons, and increased pollen production. The repercussions on human health concern both allergic asthma and rhinosinusitis, whose symptoms can be worsened by extended exposure and impacts of extreme meteorological events, such as thunderstorm asthma. In this context, traditional aerobiological monitoring techniques are still valid as staple methods, but with a rising importance of new, automatic real-time sensors and atmospheric pollen forecasting systems, which can be important for early warning and prevention, to reduce and avoid exposure to allergenic pollen during peak days and hours. Omics techniques can also give important information about sensitization and response to allergens, granting a more comprehensive picture of the effects of exposure to allergens. In this paper, we carried out a literature review to discuss all these effects, with a focus on innovative tools and preventive measures. As a result, the application of an integrated and multifactorial approach emerges as fundamental in the management of pollen-induced allergies in the context of climate change.

## 1. Introduction

Climate change (CC) and global warming are worldwide phenomena with repercussions on the environment and on human health. Anthropogenic climate change, due to its impacts, has been the object of intense investigations and has been defined with the term ‘climate crisis’ by the media [1] to underline the extension of the phenomena and the urgency they constitute.

Among the environmental effects connected to climate change are increasing temperatures and wildfires, altered distribution and characteristics of droughts, increased air pollution, floods, and extreme meteorological events such as hurricanes, thunderstorms, and sandstorms [2,3]. These events can have direct effects on human health and safety, aggravating pre-existing conditions, such as cardiovascular illnesses, and causing exposure to several risks both in life and work environments.

Environmental changes associated with CC can also affect flora and fauna, determining the spread of vector-borne diseases and changes in vegetation structure, and also favoring the spread of invasive plant species that can damage human health, for example, by producing allergenic pollen and causing the emergence of new allergies. CC can also affect plant phenology, causing an anticipation and an overall extension of the flowering season, also determining increased pollen production. In turn, a change in the vegetation distribution and characteristics may affect CC, and in particular the role played by vegetation in the carbon cycle, through modulation of photosynthesis and respiration processes (e.g., [4]).

CC is closely intertwined with air pollutants, since several pollutants can influence CC, and CC may lead to an increased production of several pollutants [3]. Chemical pollutants can also interact with biological ones (e.g., [5]), enhancing their potential to harm human health, for example by causing respiratory allergies, especially in susceptible subjects [6]. Association of pollen with natural particles, like mineral dust, may also alter allergenicity (e.g., [7]), and CC-induced modification of the intensity, pathways, and frequency of desert dust transport episodes may also modify pollen impacts. Also, projected changes in wildfire occurrence (e.g., [8]) are expected to impact pollen production and allergenicity.

Regarding the impacts of CC on occupational health, it has been proven that there is an increased risk of occupational hazards related to exposure in the workplace, mainly concerning outdoor work such as agriculture, construction, and landscaping, through exposure to heat waves, extreme meteorological events, and allergenic pollen, but also for workers in the fields of farming and veterinary medicine, who can be exposed to vector-borne diseases and emerging zoonoses [3].

The Lancet Countdown report [9] found that 12 of 20 key indicators of health threats have reached record levels, representing a growing concern for human health, with heat-related mortality having increased by 23% since the 1990s, and drought and heatwaves being associated with 124 million people facing food insecurity. Climate action, on the other hand, has proven useful in avoiding about 160,000 premature deaths.

The aim of the current review is to explore the linkages between CC and respiratory allergies, regarding both public and occupational health, with special reference to preventive actions aimed at reducing the impacts on a rapidly changing system, analyzing several literature papers. In particular, we have selected 119 studies, of which 80 were published between 2020 and 2026. To carry out this review, the main editorial browsers were used (for example, Elsevier, Springer, Nature, and Copernicus), considering the following keywords:

- Pollen forecasting system for the modeling section, considering the last 20 years.
- Climate change and pollen allergies to examine the linkage between CC and pollen, considering the most recent years.
- Aerobiological methods for the monitoring section, considering the last 20 years.

The result of this review is presented and discussed according to the following sections:

- Section 2.1 emphasizes evidence and attributions of climate change.
- Section 2.2 highlights the complexity of the impact of climate change on pollen production.
- Section 3.1 refers to climatic factors that may influence the allergic diagnosis.
- Section 3.2 focuses primarily on occupational rhinitis and mentions the role of climate change in the incidence of allergic rhinitis.
- Section 4.1 presents conventional aerobiological monitoring records alongside modern pollen databases, and Section 4.2 discusses pollen forecasting systems as actions to prevent allergies.
- Section 5 describes future projections of pollen production and the associated health impacts under climate change.

## 2. Climate Change: Up-to-Date Evidence

### 2.1. Human-Caused Climate Change

The IPCC (Intergovernmental Panel on Climate Change) Sixth Assessment Report (AR6, [10]) unequivocally states that human influence has warmed the atmosphere, ocean, and land. Global surface temperature has increased more rapidly since 1970 than during any other 50-year period in the past 2000 years. The average global temperature in 2011–2020 reached 1.1 °C above the 1850–1900 baseline, a rise that was clearly driven by human activities, primarily through the emission of greenhouse gases (GHGs) [10].

Human-induced CC has triggered rapid and widespread transformations across the atmosphere, oceans, cryosphere, and ecosystems. These changes are intensifying extreme weather events globally, causing significant damage to both natural systems and human communities.

Key climate indicators and quantified human influences are updated annually and made publicly available, reflecting the pace and scale of recent changes [11,12,13].

Carbon dioxide (CO_2_) remains the primary driver of CC, responsible for about 66% of the anthropogenic radiative forcing from all long-lived GHGs since 1750 and roughly 79% of the increase observed over the past decade. In 2024, atmospheric concentrations of CO_2_, methane (CH_4_), and nitrous oxide (N_2_O) reached their highest levels in at least 800,000 years [13,14].

In 2024, the globally averaged CO_2_ concentration was 423.9 ppm (152% of the pre-industrial level), with an increase of 3.5 ppm from 2023 (https://wmo.int/files/greenhouse-gas-bulletin-no-21 (last accessed on 4 August 2026)). This represents the largest annual rise in recent observations, exceeding the 2.4 ppm increase from 2022 to 2023 and being well above the average growth rate of 2.57 ppm yr^–1^ over the past decade [14]. The observed increase is partly attributed to the growth in total energy-related CO_2_ emissions, which rose by 0.8% in 2024 to an all-time high of 37.8 Gt CO_2_ [15].

The year 2024 was also the warmest in the 175-year observational record, with a global near-surface temperature of 1.55 °C ± 0.13 °C above the 1850–1900 average [16]. This surpasses the previous record set in 2023 of 1.45 °C ± 0.12 °C. Each year from 2015 to 2025 ranks among the eleven warmest ever recorded, underscoring the persistent and accelerating nature of global warming [13]. Besides the increase in global surface temperature, the upper 2000 m ocean heat content also reached unprecedented highs in the historical record in 2024 [17].

Paleoclimatic reconstructions and instrumental data show that GHGs began to influence warming trends with the onset of industrialization. By 1900, proxy-based temperature reconstructions already reflected this emerging trend [18]. However, other forcings, such as natural and anthropogenic aerosols, interactions with clouds, changes in land use, and complex interconnections and feedback processes have caused deviations.

### 2.2. Impact on Pollen Production

CC has been identified as a major driver of alterations in pollen production, with potential implications for allergenic diseases and ecosystems (e.g., [19,20,21,22]). Analysis of how changes in environmental parameters, including alterations in wind patterns, changes in the timing and intensity of rainfall, rising temperatures, and soil moisture, impact pollen production has revealed complex patterns. Systematic reviews published in recent years [23,24] consistently highlight a positive relationship between rising temperatures and shifts in pollen dynamics, though with important taxonomic exceptions and geographic constraints [24]. The main outcomes show that overall, warmer temperatures are associated with earlier pollen season onset, longer duration and higher pollen concentrations, which may be associated with adverse health impacts, as pollen exposure has well-known health effects in sensitized individuals [23].

In addition to changing pollen production, climate can also affect pollen release and dispersion. CC was found to affect wind patterns and wind intensity [24], which are among the most important parameters influencing pollen release and dispersal.

Precipitation patterns mediate pollen concentrations through both short-term washout effects and long-term influences on plant growth, and increase aeroallergens [23].

Increased water stress in some regions can lead to droughts that affect pollen production in both quantity and quality [25,26]. However, the overall effect of reduced water stress and increased temperature on pollen depends on the individual plant species considered and is currently being studied [27].

Evidence from some regions, such as the Mediterranean, indicates that pollen seasons have already shifted, in some cases advancing by more than a month [28,29].

Research indicates that air pollution may enhance its inflammatory potential through complex physical, chemical, and biological interactions with pollen (e.g., [30]). Allergens like pollen can attach to particulate matter with dimensions below 2.5 µm (PM_2.5_), amplifying inflammatory responses. Pollutants may also alter pollen structure, protein and lipid composition, and release timing, all of which affect allergenicity. Additionally, PM_2.5_ and ground-level ozone exert irritant effects on the respiratory tract, thereby exacerbating asthma severity [6,31].

## 3. Respiratory Allergies

Respiratory diseases, such as asthma, rhinosinusitis, and chronic obstructive pulmonary disease (COPD), can worsen due to the effects of CC and air pollution [32,33]. Among respiratory diseases, allergic pathologies play an important role: asthma affected an estimated 363 million people in 2023 and caused 442,000 deaths [34,35].

Multifactorial pathologies under genetic and environmental control, with growing socio-economic impact, are increasingly represented among the diseases that develop in the workplace. Between 30% and 40% of the world’s population suffers from some form of allergic disease (respiratory, skin) and exposure to allergens occurs in living environments and in various indoor and outdoor occupational environments [35].

### 3.1. Asthma

Allergic asthma is a chronic inflammatory lung disease characterized by breathing difficulties, coughing, and wheezing [36].

Asthma is more likely to emerge if relatives have asthma [https://www.who.int/news-room/fact-sheets/detail/asthma (last accessed on 4 August 2026)], particularly close family members, and tends to manifest in people with other allergic conditions, including eczema and rhinitis. Urbanization is associated with an increased prevalence of asthma, likely due to multiple lifestyle factors. Overweight or obese children and adults are at an increased risk of asthma. Early life events can affect lung development and increase the risk of asthma, including low birth weight, prematurity, exposure to tobacco smoke and other sources of air pollution, as well as respiratory viral infections. Exposure to a variety of environmental allergens and irritants can increase the risk of asthma, including indoor and outdoor air pollutants, house dust mites, pollen, fungi, chemicals, fumes, and dust [37,38].

Up to approximately 25% of asthma cases in adults are attributable to the work environment, particularly to allergens found primarily in the workplace [39,40].

An extreme event that must be considered in relation to pollen allergies is so-called “thunderstorm asthma” [41]. This is caused by severe thunderstorms happening during the pollen peak season, representing complex events due to numerous factors that can happen simultaneously. It causes severe and diffused cases of asthma-induced respiratory crises, attested by a large number of hospital recoveries recorded during these episodes [42,43,44].

### 3.2. Rhinosinusitis

Worldwide, a global prevalence of chronic rhinosinusitis of 5–10% and a prevalence of allergic rhinitis of 5–50% have been reported [45]. Approximately 11 million workdays are lost among people with chronic rhinosinusitis in the U.S. alone [46]. Asthma, rhino conjunctivitis, and dermatitis have tangible effects on quality of life and can reduce work productivity by up to 40% [47].

Occupational rhinitis has been described in multiple work environments and occupations due to exposure to both high-molecular weight (HMW) and low-molecular weight (LMW) agents [48]. There is typically a period of sensitization before the development of allergic rhinitis. Food handlers, for example, may become sensitized through direct transcutaneous exposure or through inhalation of aerosolized particles while handling food. Workers in food processing plants may become sensitized not only to the food they are handling, but also to additives used in the workplace, such as enzymes. Occupational rhinitis and asthma affect bakers more than the general population, with recent studies reporting incidence rates of 31% and 5%, respectively [49].

CC can have a significant impact on the incidence of allergic rhinitis. As shown in several studies, CC can influence the prevalence of allergic rhinitis as a consequence of extreme weather events, and it can also exacerbate symptom severity as an indirect result of increased pollen loads and prolonged pollen seasons [50].

## 4. Tools for Pollen Monitoring and Simulation as Actions to Prevent Allergies

### 4.1. Monitoring

Due to the negative health effects of pollen and fungal spores, monitoring stations for these allergenic biological agents have been established all over the world. Data obtained from these stations are usually published by national or international pollen monitoring networks, which provide pollen bulletins that are also useful for allergic subjects. Globally, the most common monitoring method considers the use of a Hirst-type volumetric sampler [30] and the analysis of the content of acquired samples through light microscopy, as described in the relative UNI EN norms [51,52].

Hirst-type samplers have been used to study occupational exposure to allergens, relating daily outdoor pollen concentrations to meteorological variables such as wind speed and direction, to assess main pollen sources and suggest preventive and protective measures [52]. These kinds of studies, applied to long-term, multi-year monitoring records, could give valuable information on changes in exposure levels when other environmental parameters are considered in relation to CC. This approach also makes it possible to examine measurements from workplaces with different vegetation cover, to compare the effects of different urbanization levels [53].

Traditional volumetric sampling is still the most effective and reliable approach to studying outdoor exposure to airborne allergens, also in relation to CC. Several decennial studies have shown the change in phenology of flowering allergenic plants and related it to CC [54,55]. Such data can also be used to detect the provenance of allergenic pollen grains, by comparing daily or hourly pollen concentrations with outdoor climatic variables, such as wind speed or wind direction, assessing the main sources of pollen grains, comparing monitoring data with vegetation maps, and also considering other variables such as temperature and humidity, that can have an important influence and whose dynamics are affected by CC [53,56,57]. This approach is also helpful to study airborne pollen dynamics during extreme events, like Saharan dust intrusions in Europe, that have been linked to particular pollen dynamics and have the potential to cause exposure to allergenic pollen that is not normally present in some countries or is not in flower during that particular season [58].

In addition to conventional aerobiological monitoring records, modern pollen databases may represent a valuable and still largely underexplored resource for investigating the spatial distribution of allergenic pollen sources. These datasets compile modern pollen assemblages collected from a variety of natural and artificial depositional environments, including moss polsters, surface sediment samples, forest litter, and artificial pollen traps. Although they have been primarily developed for vegetation–pollen calibration studies and pollen-based reconstructions of past environmental and climatic conditions, they may also contribute to identifying areas characterized by persistent high pollen production and dispersal from allergenic taxa, as well as improving numerical models for pollen forecasting. In this regard, the Eurasian Modern Pollen Database (EMPD) [59] and the recently established Italian modern pollen database ALIVE [60] could complement long-term aerobiological monitoring networks by providing independent information on spatial patterns of pollen deposition. The integration of modern pollen datasets with aerobiological time series may improve the characterization of areas historically subjected to elevated allergenic pollen loads and support future risk assessment and management strategies aimed at reducing allergy-related health impacts.

A new frontier in aerobiological monitoring is represented by the use of real-time automatic samplers. These instruments are very useful in detecting aerosol particles in the air, which can be identified as bioaerosol through different techniques, such as fluorescence spectroscopy, elastic scattering, holography, Raman spectroscopy, mass spectrometry, and breakdown spectroscopy [61]. Automatic detection has several advantages, the first of which is the ability to collect a large amount of data without the need for operator intervention, as well as the automatic identification of bioaerosol particles, both of which can greatly reduce the time needed to collect and process data, making monitoring much quicker and more time efficient. These characteristics can be very useful when studying the influence of meteorological variables and extreme events on the concentration of airborne pollen, giving information on the daily change in exposure levels to allergenic pollen, and acting as an effective instrument for early warning of exposure. As shown by several studies [62,63], these sensors are very effective in detecting sudden changes in bioaerosol concentration and also composition in indoor working environments, in which the presence and actions of the workers and the opening/closing of doors have been shown to have an important influence on the exposure level of workers [54,63].

Among the automatic techniques developed in the last two decades, lidar systems are of particular interest, as they allow continuous monitoring of the vertical distribution of pollen by exploiting their effect on the polarization [64,65,66] and fluorescence of the lidar signal [67,68].

On the other hand, these instruments are not capable of precise identification to the species or genus level, which is possible with a skilled operator. Thus, they provide less precise and accurate data regarding the different types of allergens that can be found in a working environment. Considering this limitation, it could be useful to operate a parallel sampling in the same environment using both a traditional volumetric sampler and a real-time sensor, to obtain a complete dataset of exposure. Table 1 shows comparative aerobiological methods for pollen evaluation.

A complete evaluation of the occupational exposome, i.e., the ensemble of the allergens to which a worker is exposed in the working environment, must not only consider the environmental exposure, but also the analysis of biomarkers of exposure and the effect in biological samples, as well as testing the individual sensitization of each worker to the allergens found in environmental samples.

There are several techniques that can be used for these kinds of analyses. To test individual sensitivity, the traditional test is the ELISA (Enzyme-Linked ImmunoSorbent Assay), which detects the presence of specific IgE antibodies in serum samples. New tests used for this kind of evaluation are multiplex microarray tests, which can simultaneously detect the sensitivity of a singular individual to many types of allergens normally found in both everyday life and working environments, such as the ISAC (ImmunoSorbent Allergo Chip), which can detect the sensitization to 112 allergens from 51 sources, and the Alex2 (Allergy Explorer 2), which can detect and quantify antibodies for more than 300 purified allergens or allergen extracts.

Another approach involves detecting microRNAs (miRNAs) in easily accessible biofluids such as blood and urine. miRNAs are increasingly recognized as promising diagnostic and prognostic biomarkers for a wide range of pathological conditions, including cancer and oxidative stress–related disorders. Notably, specific miRNA expression profiles have also been associated with allergic diseases and responses to allergen exposure [79,80,81,82].

Generally speaking, omics applications hold considerable promise for improving comprehensive evaluations of aerobiological hazards in workplace settings. Employing multiple omics platforms in parallel would provide notable benefits [83,84], particularly as new technologies now facilitate the rapid processing of extensive datasets, although issues related to financial burden and the complexity of data interpretation still persist.

Advancing omics strategies in aerobiology—such as genomics, proteomics, and metabolomics—is becoming as essential as it is in numerous areas of modern biological and medical research. Besides the financial aspects, the most significant challenges involve the integration, analysis, and eventual practical use of multi-omics information [83,84]. Nonetheless, ongoing progress in omics technologies, improvements in big data processing, and the emergence of single-cell omics innovations [85] are expected to create substantial new opportunities for research and risk characterization in occupational aerobiology.

**Figure 1:**
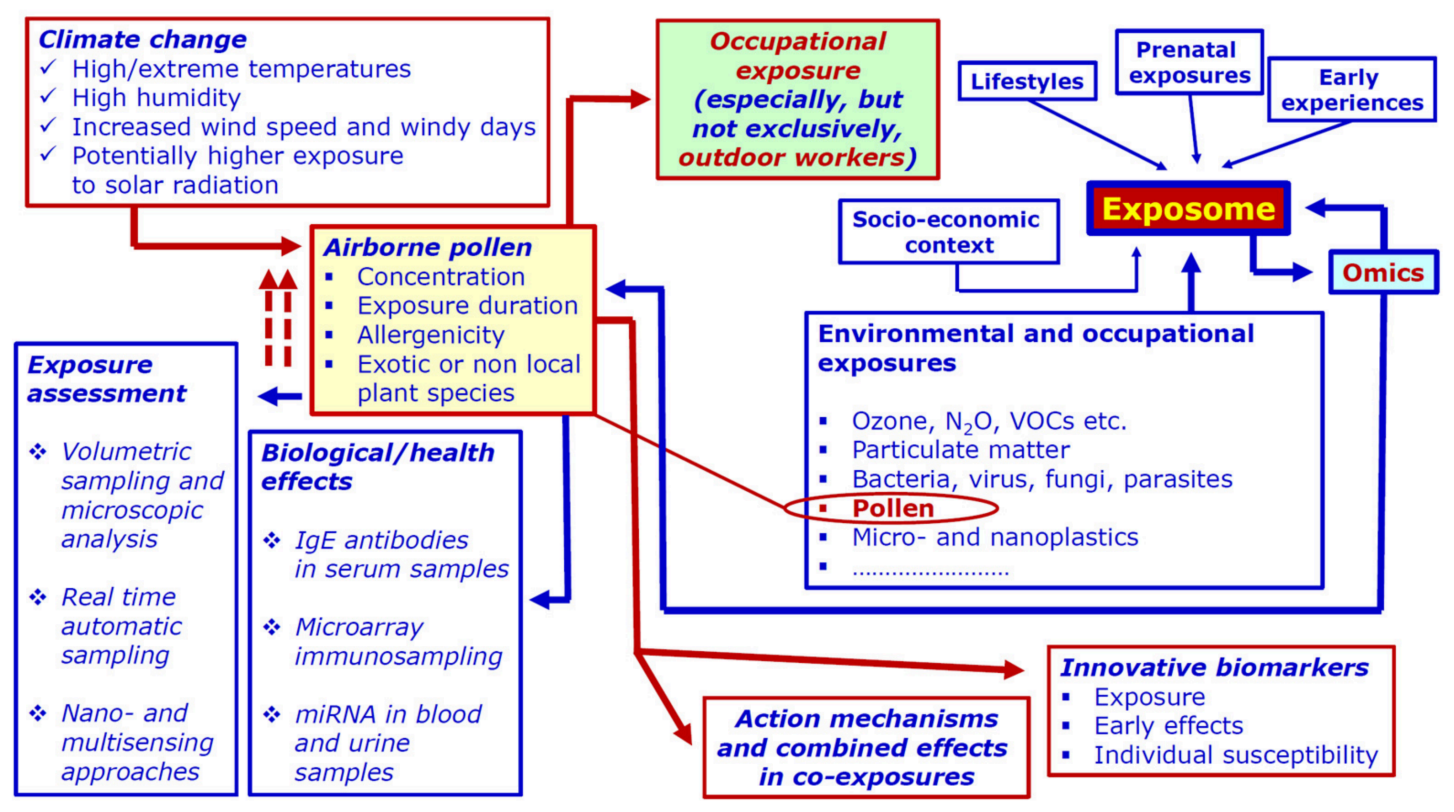
The occupational exposure to pollen and its impact on workers’ health is a concern of growing importance due to climate change. The physical consequences of a changing climate may increase pollen production and release, as well as the spread and allergenic potential of pollen grains. Occupational exposure to pollen is particularly important for outdoor workers and may be assessed by both conventional and innovative approaches. Occupational exposure to pollen is part of the so-called “exposome” (more specifically, of its subset known as the “occupational exposome”), which includes all exposures encountered by an individual from the prenatal life to death, including those due to lifestyles and social environment. The rapid development of “omics” approaches and the possibility to manage “big data” through artificial intelligence and other computational methods depict new scenarios in occupational pollen exposure assessment and management, allowing us to better define and quantify the action mechanisms at the biological level, the biological response, and the combined effects due to multiple exposures. Moreover, the possibility to identify and validate novel biomarkers of exposure, effect, and susceptibility is disclosed.

As shown in Figure 1, the use of different monitoring techniques is essential for a complete evaluation of occupational exposure and for the analysis of its complex causes and effects.

### 4.2. Models to Simulate Pollen

Atmospheric pollen simulation systems have grown substantially to support public health and allergy management. Pollen simulations rely on numerical atmospheric models adapted from chemistry transport models (CTMs), in which pollen grains are treated as passive tracers (i.e., not subjected to chemical transformations) driven by emission, transport and deposition processes. Alongside these mechanistic systems, a growing number of studies have explored data-driven approaches based on machine learning (ML) techniques. The two approaches differ substantially in terms of implementation effort, computational cost and transferability. Deterministic models are more demanding to develop, since they require an explicit description of the physical processes controlling pollen release, dispersion, transport and deposition, and simulating these processes requires computational resources. Their main strength lies in their portability: once the model has been implemented, and provided that a reliable description of taxa distribution is available, they can in principle be applied to any region of the globe. Conversely, ML-based models are much faster to run but rely on a computational expensive training phase that is generally is based on local data, which limits calibration harmonization across different systems and portability to other geographical areas.

Currently, several pollen forecasting systems have been developed and are used for both research purposes, in many cases focused on evaluating the impact of CC on pollen concentrations, and for short-term operational forecasts. WRF-Chem [86] is a mesoscale modeling system based on the Weather Research and Forecasting model (WRF; [87]) coupled with Chemistry, originally developed to simulate the concentration, transport, and dispersion of atmospheric pollutants. In [88], the coupled Weather Research and Forecasting Community Multiscale Air Quality (WRF–CMAQ) modeling system was extended with pollen emission and dispersion capabilities and applied to simulate pollen dynamics in a CC context over southern California. More recently, the National Oceanic and Atmospheric Administration (NOAA) has adopted a simplified chemistry configuration of WRF-Chem within the RAP-Chem system to generate experimental real-time forecasts of major pollen taxa across the continental U.S [89]. Beyond mechanistic models, data-driven approaches have also been applied to short-term pollen forecasting, like in [90], where the authors applied a Random Forest to predict *Quercus*, Cupressaceae, *Ambrosia* and Poaceae over the continental US; Lops et al. [91] used a deep convolutional neural network (CNN) to produce real-time 1–7-day forecasts of daily pollen counts in the Houston area for the year 2016, training the model on 2009–2015 meteorological, satellite, and processed pollen flux data.

**Figure 2:**
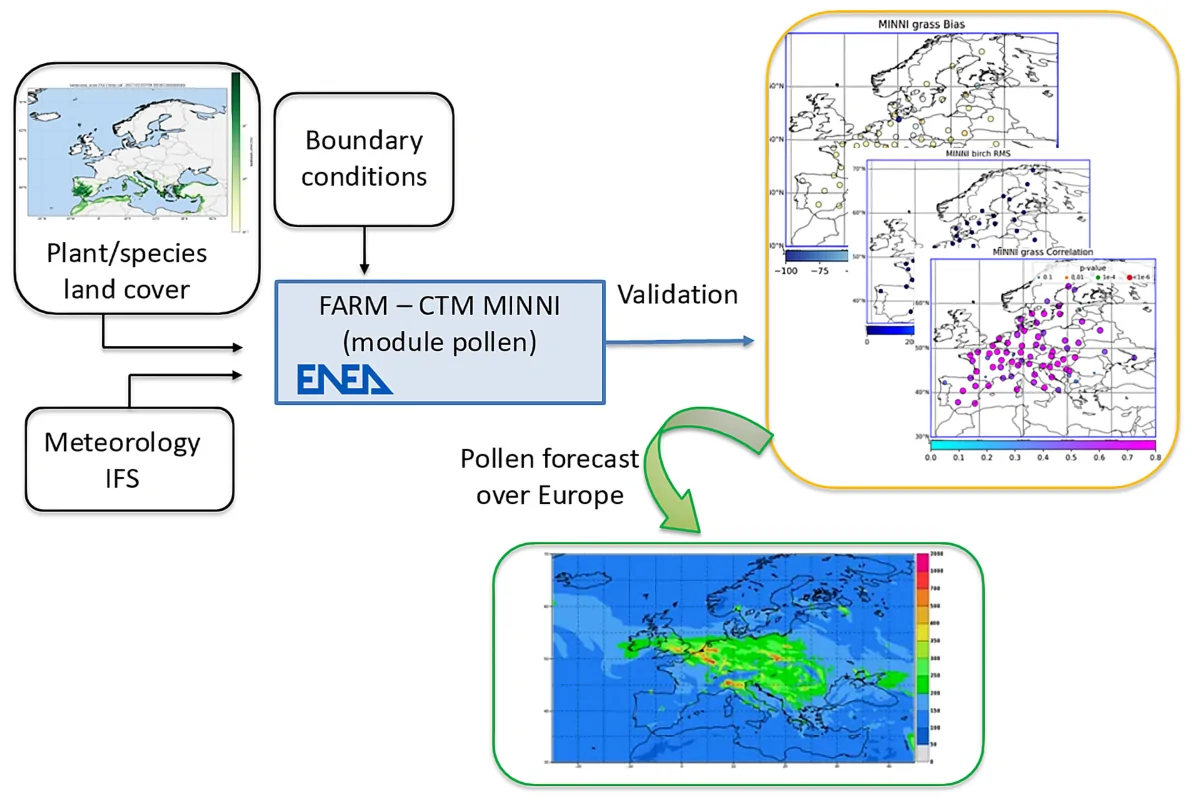
MINNI-FARM scheme of the module to produce pollen forecasts over the European domain.

In Europe, a robust air quality forecasting system has been developed within the Copernicus Atmosphere Monitoring Service (CAMS) regional system [92]. It is composed of an ensemble of 11 models providing, beyond the regulated atmospheric pollutants, pan-European forecasts at 10 km resolution for the upcoming 4 days for alder, birch, olive, ragweed, mugwort, and grass. As an example, Figure 2 shows the workflow scheme of the MINNI-FARM model [93,94,95], one member of the CAMS ensemble.

Over the same CAMS domain, Ref. [96] produced a continental reanalysis (1980–2022) for alder, birch and olive, assimilating observations from 34 countries using a 4D-Var algorithm to optimize annual emissions, delivering a consistent baseline for trend analysis and model evaluation. Other operational pollen forecasts over Europe are provided by SILAM [97,98] and ICON-ART [99]. Among research studies, [100] implemented the EMPOL 1.0 pollen emission scheme in COSMO-ART, a precursor of ICON-ART, and applied it over a European domain at 7 km resolution to simulate the 2012 birch pollen season. COSMO-ART has been also tested over complex terrain by [101] at 1.1 km horizontal resolution, to simulate birch pollen transport during the 2017 season over two Alpine mountain top sites and two lower altitude Swiss stations. Their analysis showed that pollen can be lifted above ~3000 m in the morning and advected by large-scale winds, emphasizing that topography, source location, and wind patterns strongly shape pollen distribution in complex terrain. Ref. [102] demonstrated the benefit of integrating real-time pollen observations with numerical model outputs through a real-time calibration approach, developing a method for COSMO-ART over a high-resolution domain covering the greater Alpine region, with test runs for *Alnus*, *Betula*, *Corylus* and Poaceae pollen against measurements from 13 stations.

Beyond Europe and North America, numerical pollen models demonstrate global progress. In Australia, the Victorian Grass Pollen Emissions Module (VGPEM) [103] was integrated with the ACCESS meteorological model to estimate grass pollen emissions, transport, and dispersion in the Victoria region. In Asia, examples of a combination of meteorological forecasts, statistical models and machine learning approaches can be found, particularly in regions with sparse observational networks. In Beijing, Ref. [104] used an ensemble of twelve machine learning models exploiting multi-annual pollen observations and real-time ECMWF meteorological forecasts, producing reliable short-term (1–3 day) pollen concentration forecasts. In Shanghai, Ref. [105] demonstrated the reliability of automated real-time pollen monitoring, showing good agreement with conventional Hirst-type samplers; such instruments provide high-frequency observational data that can feed data assimilation frameworks and improve forecast accuracy.

In Korea, Ref. [106] developed regression-based data-driven pollen prediction models to estimate the annual potential of seven allergenic tree taxa across nine major urban areas, using long-term Hirst-type trap data (1998–2021) combined with monthly meteorological variables. The authors showed that inter-annual variability in pollen production can be reproduced well and suggested that such annual pollen potential estimates could be combined with short-term daily forecasting systems to improve pollen predictions by accounting for inter-annual variability.

## 5. Future Projections of Pollen Production and Related Health Impacts

The consequences of CC, such as rising global temperatures and altered meteorological patterns, are expected to modify the distribution and abundance of allergenic pollen, as well as extending the duration of pollen seasons, thereby increasing allergen exposure and exacerbating allergic conditions.

Predictive models based on bioclimatic variables have revealed that CC will further lead to an increased incidence of allergies as a result of higher pollen counts from some important pollen species. However, existing predictive models focus on some regions of the Northern Hemisphere, such as Europe and North America, while global estimates are not available.

A recent study focusing on the Milan area examines the correlation of meteorological variables (temperature, relative humidity, precipitation, wind, and solar radiation) and the daily concentration of airborne pollen from herbaceous taxa with high allergenic potential in Europe (*Ambrosia*, *Artemisia*, Cannabaceae, Chenopodiaceae/Amaranthaceae, Poaceae, and Urticaceae) during the 1995–2022 period [28]. Various seasonal pollen descriptors have been analysed, such as the starting day, the ending day, the duration, and the number of days within a season with a concentration value greater than 1 p/m^3^. Temperature has been shown to be the primary driver of phenological changes, while the effects of precipitation are more complex to assess, as benefits and detriments for plant growth depend on the species’ ecology. The observed trends have been used as base for estimating hypothetical scenarios of pollen levels for the different species on temporal horizons of 20, 40, and 60 years into the future. Based on the current linear trend in temperature, both the Urticaceae and the Poaceae pollen season are predicted to start earlier and have a longer duration. The same is predicted for *Artemisia* and *Ambrosia*, but with a smaller increase in duration.

Singh and Kumar [107] presented the results of a study aimed at predicting the distribution of three ragweed species in Europe by means of a model using the MAXENT (v3.3.3k) software. The model was run with two IPCC CC scenarios (the moderate RCP 6.0 and the high RCP 8.5 GHGs emission scenarios), and the output estimates that by the year 2100, the distribution range of all three ragweed species will increase towards Northern and Eastern Europe under all climate scenarios, and Europe will be affected by severe ragweed-associated allergy problems, potentially affecting millions of people.

Ragweed pollen allergy is projected to become a common health problem across much of Europe, and sensitization to ragweed will more than double, increasing from the current total of 33 million to 77 million people by 2041–2060, according to projections by [108], with the greatest proportional increase in countries where sensitization is now relatively uncommon (e.g., Germany, Poland, France). The projections are based on two different suites of regional climate/pollen models, two IPCC GHGs emission scenarios RCP 4.5 and 8.5, and three different plant invasion scenarios.

Other plants, like birch trees (*Betula* spp.), which are important sources of aeroallergens in Central and Northern Europe, may experience a decrease at lower altitudes, with a consequent reduction in the amount of airborne birch pollen, but may expand at high altitudes because of CC [109].

A platform for large-scale airborne pollen modeling was developed by [110] and used to simulate the spatiotemporal distributions of major pollen aeroallergens, such as oak and ragweed pollen, across the contiguous United States for the specific IPCC RCP 8.5 scenario, considering changes in temperature, precipitation, humidity, and wind. The results show that the ragweed pollen season will start earlier and last longer for all the nine climate regions, with increasing average pollen concentrations in most regions. The response of the oak pollen season to CC varies across the nine climate regions, with the largest increase in pollen concentration predicted for the Northeast region.

Zhang and Steiner [111] developed a continental-scale model to project how CC will alter future pollen emissions across the United States. Using the Pollen Emissions model for Climate Models (PECM), driven by CMIP6 climate projections, the authors simulate future pollen season timing and total emissions for 13 major allergenic taxa. The results show a shift in pollen season phenology, driven by increasing temperatures, with spring-flowering taxa projected to initiate pollen release 10–40 days earlier by the late century (2081–2100) compared to the historical period (1995–2014), while the peak emission of summer and autumn taxa is delayed by 5–15 days, and the overall pollen season is lengthened, producing greater temporal overlap among allergenic species.

Future climate conditions also significantly enhance pollen production. Under temperature- and precipitation-driven changes alone, the annual pollen emissions are expected to increase by approximately 16–40% relative to the historical period. When accounting for CO_2_-induced fertilization effects, simulated emissions rise dramatically, potentially reaching up to a 200% increase in total pollen load.

Weber [112] reviewed how rising atmospheric CO_2_ concentrations and global temperature are reshaping the production, seasonality, and distribution of airborne allergens such as pollen and fungal spores. The paper synthesizes observational data, experimental findings, and modeling studies to assess both current trends and expected future developments. Almost all the studies quoted in the review are from the Northern Hemisphere (Europe and USA), with a single Australian study. CC has broadly led to earlier onset of pollen seasons for many allergenic tree species, grasses, and weeds, as well as to a lengthening of the overall duration of pollen seasons and enhanced pollen biomass per plant, thus resulting in greater annual pollen loads at the population level. Finally, higher temperatures and changes in precipitation patterns facilitate the northward and altitudinal expansion of allergenic plant species and the establishment of invasive allergenic weeds in previously unsuitable climates. The already measurable effects of CC will likely amplify the burden of allergic respiratory diseases in the coming decades.

Recently, the major findings of the previous papers were summarized by [113], in which the authors confirmed how CC affects aeroallergen biology, human exposure, and co-exposure with air pollutants, as well as the potential consequences for allergic disease incidence, severity, and distribution across populations. Not only trees, but also grasses, weeds, and other allergenic species show changes in phenology (the timing of flowering and pollen release), which may translate into prolonged periods of allergen exposure. The co-exposure to elevated aeroallergen loads and pollutants is associated with increased risk of asthma onset, exacerbations, allergic rhinitis, and higher morbidity in allergic diseases, particularly for vulnerable populations, such as children, the elderly, and economically or socially marginalized groups. There are still gaps and uncertainties in predicting the exact burden of future allergic disease because of complex interactions among climate variables, pollutant emissions, plant biology, urbanization, and social determinants. Forecasting models, up to now developed using regressions, may benefit from the use of deep neural network models.

In their editorial, Aguilera et al. [114] highlighted potential strategies to mitigate CC’s impact on allergic diseases, which include understanding the specific environmental factors influencing allergenicity and a multidisciplinary approach to addressing CC’s impact on allergic diseases, foreseeing collaboration among immunologists, allergists, environmental scientists, and public health experts to develop comprehensive strategies for prevention and mitigation. Singh and Kumar [107] pointed out how the capacity to anticipate a future allergic disease burden can help public health agencies in planning strategies to mitigate the health challenges expected in future years, in addition to informing and preparing the population on the risk of rhinitis and asthma exacerbation during pollen season.

## 6. Discussion and Conclusions

The impact of climate change on pollen production, seasonality, and concentration deserves particular attention in both public and occupational health. The health impacts are now being examined taking into account combined effects of different factors [115], including occupational exposure to solar radiation [116], the burden of vector-borne diseases among working populations [117], and the rise of work-related allergic conditions [118]. In this framework, the management of pollen allergies requires an integrated and multifactorial approach: traditional aerobiological monitoring should be complemented by new automatic real-time technologies and forecast models to accurately investigate the long-term change and variability of pollen levels driven by climate change. Moreover, molecular approaches, including omics techniques, together with computational methods such as artificial intelligence, enable the elucidation of underlying biological mechanisms, patients’ responses, and the complex effects of multiple exposures. Furthermore, it is essential to strengthen health impact communication through targeted education campaigns addressing pollen exposure and climate change. This includes implementing early warning systems and promoting data sharing and best practices, especially for susceptible workers and vulnerable populations.

Effective information and communication play a crucial role in preventing issues related to emerging health hazards: public education, worker training, and the development of more effective measures to reduce pollution and mitigate the effects of CC are critically needed worldwide.

In addition to promoting a multidisciplinary framework and personalized interventions to optimize the management of occupational exposure to pollen, and emphasizing the importance of early detection of effects as well as the management of pollen-related diseases, it is important to encourage a large, multi-level discussion on these topics involving experts and stakeholders. Moreover, the importance of structural measures to reduce environmental and occupational exposure to pollen must be stressed. Urban settings are critical in this regard. The appropriate management (e.g., controlling, avoiding or removing allergenic plant species) of existing urban green or blue areas (parks, gardens, trees along the streets, little lakes and basins), as well as the implementation of both green and blue areas in the urban texture, are of particular importance. It must also be kept in mind that green and blue areas in an urban context contribute to mitigating the so-called “heat island” effect, which is worsened by the changing climate. Moreover, they contribute to improving the adaptation measures to climate change at individual and community levels, ensuring, at the same time, many co-benefits for both physical and mental health.

The widespread adoption of automatic real-time sensors is essential for the continuous monitoring of environmental conditions, enabling early warning signals in occupational settings and supporting the large-scale analysis of bioaerosol dynamics in indoor and outdoor environments. Current evidence in the literature highlights the key role of artificial intelligence (AI) [119] in strengthening occupational health and safety through advanced sensors and biosensors capable of estimating real-time exposure to environmental hazards, including airborne pollen and fungal spores, thereby improving allergy surveillance and management. AI-enabled wearable devices and mobile applications integrate real-time environmental parameters such as pollen levels, airborne pollutants, humidity, and temperature with individual clinical data to support personalized prevention strategies. In parallel, the integration of multi-omics technologies with big data analytics and advanced bioinformatics algorithms is continuously enriching open-access databases that support intelligent sensing platforms. Therefore, in aerobiology, the combined application of AI-driven real-time sensors and volumetric samplers enables more accurate detection and classification of allergenic bioaerosols, while improving exposure assessment, risk characterization, and the real-time management of allergic diseases in occupational and environmental contexts [35]. However, their implementation may be hindered by the high costs associated with equipment acquisition, maintenance, and calibration, as well as by the need for specialized technical support, creating a disparity in the diffusion of these networks among countries. Furthermore, the complementary deployment of operational pollen forecast models alongside real-time monitoring systems represents a powerful synergy for effective early warning systems. In such a context, forecast models provide spatiotemporal predictions of pollen concentrations that can anticipate peak events, while observational data continuously feed back into model evaluation and refinement, e.g., enabling the identification of deficiencies in parameterized processes; forecast skill can be further improved by integrating real-time observations with numerical model outputs through calibration approaches. Collectively, these findings underline the central aim of this work: to promote a shift toward an integrated, multidisciplinary framework bridging real-time and advanced aerobiological monitoring strategies, molecular and multi-omics platforms, and digital technologies to improve the understanding and management of pollen-related diseases in the face of rapid climate change. Such an approach is no longer optional, but crucial to translate scientific innovations into effective and actionable solutions, informing timely decision-making, optimizing risk stratification, and advancing the development of personalized interventions to protect susceptible and vulnerable populations.

## Figures and Tables

**Table 1:** Summary of the comparative aerobiological methods used for pollen evaluation. The table is based on References [53,61,63,69,70,71,72,73,74,75,76,77,78].

Method	Advantages	Disadvantages	Response Time	Technical Notes
Automatic Hirst-type volumetric sampler	Based on standardized norms, making pollen data comparable	The presence of a skilled operator is required. Labor-intensive sample analysis	Several hours to days for preparation, evaluation and calculation, depending on sample size	Able to operate automatically for up to seven days. Remains the reference method for routine aerobiological monitoring
Rotorod sampler	Ease of use, high efficiency for pollen and larger fungal spores. Efficiency is comparable to Hirst-type samplers for particles > 10 μm	Rotating arm impactors are not recommended for sampling particles < 10 μm. The presence of a skilled operator is required	Several hours for preparation, evaluation and calculation, depending on sample size	Is still a relevant sampling method, mainly used in the United States of America
Molecular techniques	High sensitivity and specificity	Limited by the need for availability of specific sequence primers	Hours to days, depending on the time needed for DNA extraction, amplification and analysis	Availability of specific sequence primers is required. Sensitivity and specificity need to be tested for each target
Immunochemical assays (ELISA: enzyme-linked immunosorbent assay, IFA: immunofluorescence assay)	Recognizes allergens on airborne particles through color or fluorescence	Quali/quantitative methods need to be standardized	Several hours	Detects allergens, but does not directly identify or quantify pollen grains
Real-time automatic samplers	Able to operate through continuous automated sampling, with high levels of automation and reliability	The identification of different biocomponents is not as accurate as other methods	The data is recorded in real time	Novel frontier in aerobiological monitoring but still under development, requiring accurate interpretation of the processed data
Nuclear magnetic resonance (NMR)	Applicable to metabolites extracted from both airborne pollen and pollen mixtures. Able to provide valuable information on metabolites and chemical profiles of pollen grains	The method needs to be optimized for the chemical characterization and identification of metabolite groups	Hours to days, depending on the sample	This method is not available in all laboratories and needs qualified personnel
Environmental DNA (eDNA)	Can identify multiple pollen types at the same time. Gives a good picture of diversity in aerobiological samples	Difficult interpretation. Needs to be standardized	Hours to days, depending on the sample	This approach needs a multidisciplinary expertise

## Data Availability

No new data were created or analyzed in this study.

## Abbreviations

The following abbreviations are used in this manuscript:

CC	Climate change
GHG	Greenhouse gas
COPD	Chronic obstructive pulmonary disease
